# Cocaine Withdrawal Causes Delayed Dysregulation of Stress Genes in the Hippocampus

**DOI:** 10.1371/journal.pone.0042092

**Published:** 2012-07-30

**Authors:** M. Julia García-Fuster, Shelly B. Flagel, S. Taha Mahmood, Stanley J. Watson, Huda Akil

**Affiliations:** 1 University Research Institute on Health Sciences, University of the Balearic Islands, Palma de Mallorca, Spain; 2 Department of Psychiatry, University of Michigan, Ann Arbor, Michigan, United States of America; 3 Molecular and Behavioral Neuroscience Institute, University of Michigan, Ann Arbor, Michigan, United States of America; University of Regensburg, Germany

## Abstract

Relapse, even following an extended period of withdrawal, is a major challenge in substance abuse management. Delayed neurobiological effects of the drug during prolonged withdrawal likely contribute to sustained vulnerability to relapse. Stress is a major trigger of relapse, and the hippocampus regulates the magnitude and duration of stress responses. Recent work has implicated hippocampal plasticity in various aspects of substance abuse. We asked whether changes in stress regulatory mechanisms in the hippocampus may participate in the neuroadaptations that occur during prolonged withdrawal. We therefore examined changes in the rat stress system during the course of withdrawal from extended daily access (5-hours) of cocaine self-administration, an animal model of addiction. Tissue was collected at 1, 14 and 28 days of withdrawal. Plasma corticosterone levels were determined and corticosteroid receptors (GR, MR, MR/GR mRNA ratios) and expression of other stress-related molecules (HSP90AA1 and HSP90AB1 mRNA) were measured in hippocampal subfields using *in situ* hybridization. Results showed a delayed emergence of dysregulation of stress genes in the posterior hippocampus following 28 days of cocaine withdrawal. This included increased GR mRNA in DG and CA3, increased MR and HSP90AA1 mRNA in DG, and decreased MR/GR mRNA ratio in DG and CA1. Corticosterone levels progressively decreased during the course of withdrawal, were normalized following 28 days of withdrawal, and were correlated negatively with GR and positively with MR/GR mRNA ratio in DG. These results suggest a role for the posterior hippocampus in the neuroadaptations that occur during prolonged withdrawal, and point to a signaling partner of GR, HSP90AA1, as a novel dysregulated target during cocaine withdrawal. These delayed neurobiological effects of extended cocaine exposure likely contribute to sustained vulnerability to relapse.

## Introduction

Cocaine withdrawal has been associated with negative emotional states such as elevations in brain reward thresholds and increases in anxiety-like behavior and depressive-like states (reviewed in [1]). Identifying key substrates for such negative emotional states will help ascertain the underlying neurobiology of cocaine withdrawal. In fact, the long-lasting molecular neural adaptations within the mesolimbic dopamine system [2] induced by chronic cocaine self-administration (SA) and subsequent withdrawal are thought to underlie cocaine addiction and contribute to sustained vulnerability to relapse [3]–[5]. Emerging evidence has broadened our view of the neural substrates implicated in addiction beyond that of the mesolimbic reward system, showing drug-induced neuroplastic changes outside of the classical dopamine circuitry [6]. In particular, the hippocampus has received increasing attention for its role in addiction and relapse [7]. For example, suppression of hippocampal neurogenesis enhances resistance to extinction of drug-seeking behavior [8]. Moreover, we have shown drug-induced alterations in hippocampal gene expression [9] and phenotypic differences in adult hippocampal neurogenesis in rats that are more or less prone to addictive behavior [10].

Given its neural connections with the reward system [11], [12], and role in mediating the stress response, it is reasonable to suspect that the hippocampus plays a major role in addictive behaviors. Indeed, stress is a major trigger of drug abuse and relapse, and the hypothalamic-pituitary-adrenal (HPA) axis –the stress axis– is directly modulated by the hippocampus. Upon activation of the HPA-axis, corticosterone (CORT) is released from the adrenal gland and then exerts its biological effects by binding to mineralocorticoid receptors (MR) and/or glucocorticoid receptors (GR). These two receptors act in a complementary fashion to regulate the stress response. MR is the high affinity, low capacity receptor, operative at low CORT concentrations, whereas GR is the low affinity, high capacity receptor and binds excess glulcocorticoids, particularly in the hippocampus, which contains the highest concentration of GR [13], [14]. Hippocampal GR activation inhibits activity of the HPA axis and helps to maintain appropriate levels of hormone via neural connections to the hypothalamus [15].

Like stress, cocaine stimulates the release of CORT in rats [16] and glucocorticoid receptors are thought to mediate the reinforcing effects of the drug [17]. For example, selective inactivation of the GR gene in the brain of mice decreases initial drug-seeking behavior by flattening the dose-response function for cocaine SA and suppressing sensitization [17]. Moreover, work from our group has shown overexpression of GR in the forebrain of mice early in life increases both anxiety and cocaine sensitization in adulthood [18]. Further, glucocorticoid hormones have been described to facilitate acquisition, maintenance and relapse of cocaine SA [19], [20] and it has been suggested that reductions in basal CORT levels following cocaine SA might be driven by a concomitant increase in hippocampal GR protein [21].

Against this background, we asked whether changes in stress regulatory mechanisms in the hippocampus participate in the neuroadaptions that occur during prolonged withdrawal. To do so, we relied on an animal model that captures some of the key features of human cocaine addiction, including a progressive escalation of cocaine intake [22]. We therefore examined changes in the following molecules during the course of withdrawal (Day 1, Day 14, Day 28) from extended daily access (5 hours) cocaine SA: (1) plasma CORT levels; (2) corticosteroid receptors mRNA (GR, MR, MR/GR ratio) and (3) other stress-related genes that are part of the GR signaling pathway (HSP90AA1 mRNA, HSP90AB1 mRNA) in the hippocampal subfields (DG, CA regions).

## Materials and Methods

### Extended Access (LgA) Cocaine Self-Administration (SA)

We used hippocampal sections from rats behaviorally characterized in a recently published study [9]. Procedures followed the ethical guidelines of the University of Michigan Committee on Use and Care of Animals (UCUCA). This study was reviewed and approved by UCUCA. Forty-eight adult male Sprague-Dawley rats (Charles River Laboratories, Wilmington, MA) underwent jugular catheterization surgeries. After recovery, rats were trained to SA cocaine (FR-1), and then allowed LgA (5 h-session/day) to cocaine (0.5 mg/kg/infusion) for 14 days. Following the last SA session, LgA rats were assigned to groups that were counterbalanced by drug intake and underwent withdrawal for 1, 14, or 28 days (LgA-Day 1, n = 7; LgA-Day14, n = 7; LgA-Day 28, n = 6). No-drug (ND) controls, which also underwent jugular catheterization surgeries, were taken to the same test room and placed in boxes similar to operant chambers. Following the last session, ND rats were left in their home cage for 1, 14 or 28 days (ND-Day 1, n = 6; ND-Day 14, n = 5; ND-Day 28, n = 6).

### Tissue Collection

Rats were killed by rapid decapitation at 1, 14 or 28 days of withdrawal following the final SA session. The left half-brain was frozen in −30°C isopentane and stored at −80°C. For each rat, a series of every 8^th^ section was cryostat-cut at 30 µm and slide-mounted throughout the entire extent of the hippocampus (−1.80 to −6.80 mm from Bregma) and kept at −80°C until further analysis.

### Corticosterone (CORT) Levels

Trunk-blood was collected in heparin-coated tubes at the assigned withdrawal period under “basal” conditions (between 10 am and 12 pm). Blood was processed for plasma CORT measurements by a radioimmunoassay from MP Biomedicals (Orangeburg, NY).

### *In Situ* Hybridization (ISH) Analysis

ISH was used to detect changes in corticosteroid receptors (GR, MR, MR/GR) and heat shock proteins (HSP90AA1, HSP90AB1) at mRNA level in DG and CA regions (CA1, CA2, CA3) throughout the whole extent of the hippocampus (Bregma −1.80 to −6.30 mm) during cocaine withdrawal (1, 14 or 28 days). Each stress marker was analyzed in one series (∼24 sections) of hippocampal tissue. Before probe hybridization, tissue sections (30 µm) from the left half-brain were fixed in 4% PFA at room temperature, rinsed with salt buffers, and dehydrated with graded alcohols. After air-drying, sections were hybridized with a ^35^S-labeled cRNA probe previously cloned from cDNA fragments with specific primers using standard *in vitro* transcription methodology (GR: 402 nucleotide fragment directed against the rat GR mRNA coding region, nucleotides 765–1167; MR: 327 nucleotide fragment directed against the rat MR mRNA coding region, nucleotides 4260–4587; HSP90AA1∶454 nucleotide fragment directed against the rat HSP90AA1 mRNA coding region, nucleotides 318–772; HSP90AB1∶564 nucleotide fragment directed against the rat HSP90AB1 mRNA coding region, nucleotides 1245–1787). Probes were labeled by incorporation of ^35^S-UTP and ^35^S-CTP and hybridized to tissue overnight at 55°C. Sections were then washed with increasing stringency, dehydrated with graded alcohols, air-dried, and depending on the probe of interest exposed to film for one day to two weeks. Specificity of hybridization signal was confirmed for each probe with a sense probe control (data not shown). Digital images were scanned and integrated optical density (IOD) was measured in hippocampus DG and CA regions (CA1, CA2, CA3) for each rat using an image analysis system (Image J, Version 1.43u). Values obtained represent average of measurements taken from up to 24 sections per animal. Quantitative analyses incorporating distance from Bregma (−1.80 mm to −6.30 mm) were also conducted and represented as mean IOD at each specific Bregma level [23].

### Data and Statistical Analysis

SA behavior was analyzed using linear mixed effects model analysis (PASW 18.0) followed by Bonferroni corrected *post-hoc* comparisons [9]. The neurobiological data was analyzed using one-way or two-way ANOVAs (GraphPad Prism, Version 5) followed by Bonferroni corrected *post-hoc* comparisons or Student’s one-tailed *t*-test when appropriate. Results are expressed as mean values ± SEM. Pearson’s correlation coefficients were calculated to test for significant relationships between variables. The level of significance was *p*≤0.05.

## Results

### Escalation of Cocaine Intake during Cocaine SA Procedures

**Figure 1:**
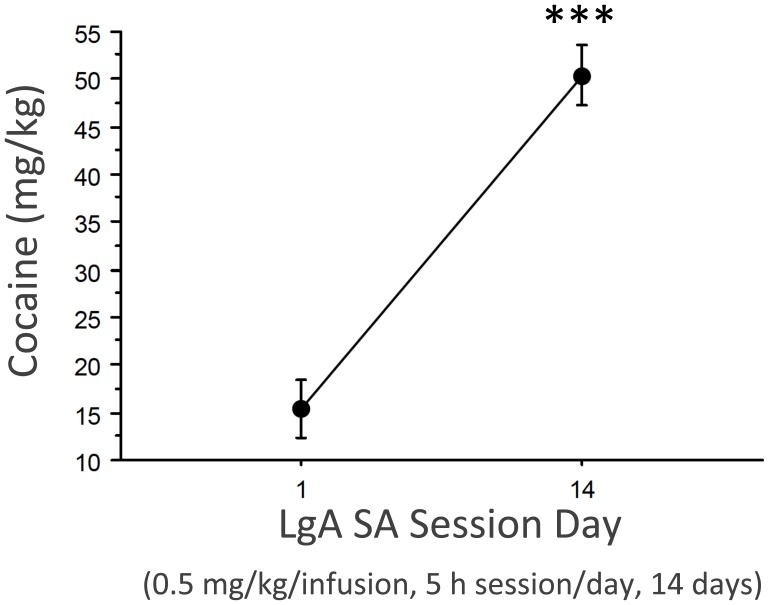
Escalation of cocaine intake during LgA cocaine SA procedures. Data represent mean±SEM amount of cocaine intake (0.5 mg/kg cocaine per infusion) over the entire daily sessions (5-h session per day of cocaine SA for 14 sessions) for session day 1 vs. day 14, ***p<0.001.

As show in Figure 1 (adapted from [9]), LgA cocaine SA procedures progressively increased daily cocaine intake from an average of 15 mg/kg (day 1) to 50 mg/kg (day 14). Thus, this served as a good model to examine the effects of withdrawal on hippocampal stress molecules.

### Basal CORT

**Figure 2:**
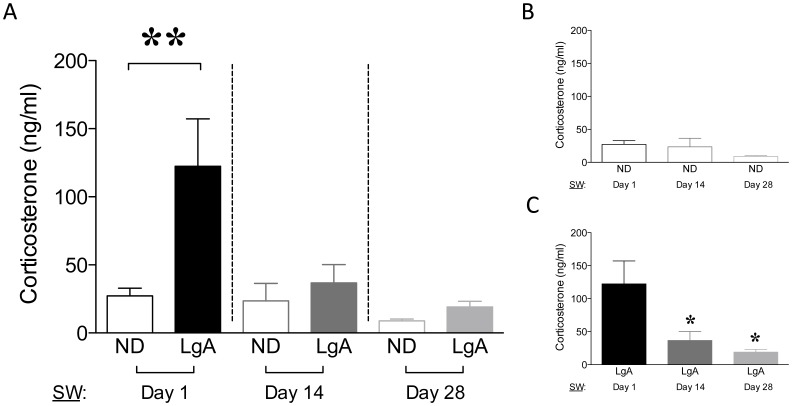
Plasma corticosterone (CORT) levels. (A) Basal CORT (ng/ml). (B) ND-groups. (C) LgA-groups. Data are expressed as mean±SEM, **p<0.01; *p<0.05.

There was a significant effect of group (ND vs. LgA; F_1,31_ = 6.956, p<0.05), withdrawal time-point (Day 1–14–28; F_2,31_ = 6.079, p<0.01), and a group x time-point interaction (F_2,31_ = 3.564, p<0.05) for CORT levels. There was an increase in CORT on Day 1 of withdrawal (p<0.01, Figure 2A) relative to the other two withdrawal time-points. Further, there was a time-dependent decrease among LgA groups (F_2,17_ = 5.896, p<0.05 vs. Day 1, Figure 2C), but no differences in CORT among control groups (Figure 2B). Taken together, these results suggest that the enhanced HPA response mediated by cocaine 24 h after the last SA session (Day 1) normalized with the course of withdrawal.

**Figure 3:**
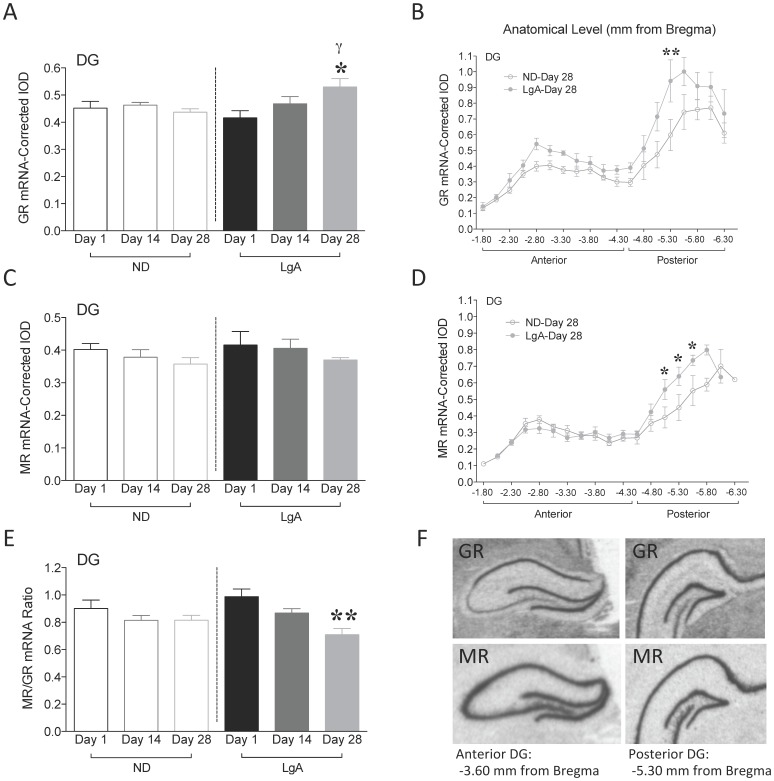
Corticosteroid receptors gene expression in DG. (A) GR mRNA. γp<0.05 vs. ND-Day 1; *p<0.05 vs. LgA-Day 1. (B) GR mRNA by anatomical level at Day 28 of cocaine withdrawal. **p<0.01. (C) MR mRNA. (D) MR mRNA by anatomical level at Day 28 of cocaine withdrawal. *p<0.05. (E) MR/GR mRNA ratio. **p<0.01. (F) Representative X-ray image of GR and MR mRNA in anterior (−3.60 mm from Bregma) and posterior DG (−5.30 mm from Bregma). Data are expressed as mean±SEM.

### Delayed Dysregulation of Stress-Genes in Hippocampus

#### Corticosteroid receptors: GR and MR mRNA

GR mRNA was analyzed in the DG of rats during the course of cocaine withdrawal. There was no effect of group or withdrawal time-point, but a significant group x time-point interaction (F_2,31_ = 3.670, p<0.05, Figure 3A). *Post-hoc* analysis revealed an increase in DG GR mRNA at Day 28 of withdrawal (ND-Day 28 vs. LgA-Day 28, p<0.05). There were no differences in GR mRNA among control groups. When comparing LgA groups, GR mRNA was increased at Day 28 relative to Day 1 (F_2,17_ = 4.042, p<0.05). When Bregma level was taken into consideration, at Day 28 there was an effect of group (F_1,186_ = 33.13, p<0.001, Figure 3B) and anatomical level (F_18,186_ = 25.76, p<0.001), but no interaction. *Post-hoc* comparisons revealed that GR mRNA changes occurred in posterior DG (distance from Bregma: −5.30 mm, p<0.01). Anatomical level was taken into consideration at Day 1 and Day 14 of withdrawal and although there were no significant group differences observed, there was evidence for increased GR mRNA in posterior DG (distance from Bregma: −4.80 mm, p<0.05, data not shown) at Day 14. These results indicate a progressive increase in GR mRNA in posterior DG during the course of cocaine withdrawal.

MR mRNA was also analyzed in the DG. There was no effect of group, withdrawal time-point, and no group x time-point interaction (Figure 3C). There were no differences in MR mRNA among control, or LgA groups. When Bregma level was considered at Day 28, there was an effect of group (F_1,153_ = 7.556, p<0.01), anatomical level (F_16,153_ = 24.45, p<0.001), and an interaction (F_16,153_ = 2.206, p<0.01, Figure 3D). *Post-hoc* comparisons revealed an increase in MR mRNA in posterior DG (distance from Bregma: −5.05 mm, p<0.05; −5.30 mm, p<0.05; −5.55 mm, p<0.05). There were no effects of anatomical level at Day 1 or Day 14 of withdrawal (data not shown). These results show the emergence of a delayed increase in MR mRNA in posterior DG following cocaine withdrawal.

**Table 1:** GR, MR, MR/GR, HSP90AA1 and HSP90AB1 mRNA in CA subfields of the hippocampus.

GR mRNA-Corrected IOD										
	ND-Day 1	ND-Day 14		ND-Day 28	LgA-Day 1		LgA-Day 14		LgA-Day 28	
**CA3**	0.216±0.013	0.202±0.012		0.209±0.015	0.189±0.009		0.228±0.013		0.244±0.011, *	
**CA2**	0.052±0.004	0.050±0.002		0.051±0.008	0.053±0.003		0.062±0.006		0.064±0.005	
**CA1**	0.514±0.034	0.512±0.017		0.472±0.013	0.474±0.018		0.524±0.028		0.555±0.029	
**MR mRNA-Corrected IOD**										
	**ND-Day 1**	**ND-Day 14**		**ND-Day 28**	**LgA-Day 1**		**LgA-Day 14**		**LgA-Day 28**	
**CA3**	0.297±0.016	0.266±0.010		0.270±0.017	0.296±0.018		0.302±0.015		0.278±0.019	
**CA2**	0.204±0.005	0.165±0.008		0.170±0.022	0.191±0.010		0.209±0.012		0.187±0.017	
**CA1**	0.499±0.021	0.484±0.024		0.442±0.024	0.503±0.037		0.472±0.033		0.444±0.029	
**MR/GR mRNA Ratio**										
	**ND-Day 1**	**ND-Day 14**		**ND-Day 28**	**LgA-Day 1**		**LgA-Day 14**		**LgA-Day 28**	
**CA3**	1.445±0.073	1.358±0.126		1.342±0.044	1.583±0.074		1.621±0.195		1.186±0.110	
**CA2**	4.623±0.391	4.209±0.244		4.598±0.536	4.795±0.405		4.674±0.647		4.248±0.345	
**CA1**	1.275±0.066	1.164±0.090		1.061±0.060	1.333±0.121		1.117±0.019		0.948±0.080, *	
**HSP90AA1 mRNA-Corrected IOD**										
	**ND-Day 1**		**ND-Day 28**			**LgA-Day 1**		**LgA-Day 28**		
**CA3**	0.243±0.014		0.235±0.015			0.229±0.017		0.203±0.014		
**CA2**	0.027±0.003		0.023±0.003			0.026±0.001		0.025±0.001		
**CA1**	0.142±0.010		0.140±0.007			0.137±0.010		0.124±0.007		
**HSP90AB1 mRNA-Corrected IOD**										
	**ND-Day 1**		**ND-Day 28**			**LgA-Day 1**		**LgA-Day 28**		
**CA3**	0.253±0.010		0.295±0.011			0.267±0.020		0.260±0.008		
**CA2**	0.030±0.001		0.028±0.003			0.029±0.003		0.028±0.001		
**CA1**	0.208±0.008		0.214±0.013			0.200±0.012		0.223±0.012		

Data are expressed as mean±SEM mRNA level (corrected IOD). *p<0.05 when comparing LgA-Day 1 vs. LgA-Day 28.

The balance in MR- and GR-mediated effects (MR/GR ratio) in hippocampus appears to be critical for neuronal excitability, stress responsibility, and behavioral adaptability. Thus, we analyzed MR/GR ratios. There was no effect of group, but a significant effect of withdrawal time-point (F_2,31_ = 7.900, p<0.01), and no group x time-point interaction (Figure 3E). There were no differences among control groups. When comparing LgA groups, MR/GR ratio was decreased at Day 28 (F_2,17_ = 9.166, p<0.01 vs. Day 1). This suggests that the magnitude of GR mRNA increase is higher relative to MR mRNA at this time point.

GR, MR and MR/GR mRNA levels were also quantified in CA hippocampal subfields (Table 1). There were no differences between groups in these subregions at any time-point of withdrawal. However, when comparing only LgA groups, CA3 GR mRNA was increased at Day 28 (F_2,17_ = 6.256, p<0.001, *post-hoc* test, p<0.05 vs. LgA-Day 1, Table 1). This change paralleled the increase in DG GR mRNA at Day 28. Furthermore, CA1 MR/GR ratio was decreased at Day 28 (F_2,17_ = 4.992, p<0.05, *post-hoc* test, p<0.05 vs. LgA-Day 1, Table 1). This change was in the same direction as the decrease in DG MR/GR mRNA ratio at Day 28.

#### HSP90AA1, HSP90AB1 mRNA

**Figure 4:**
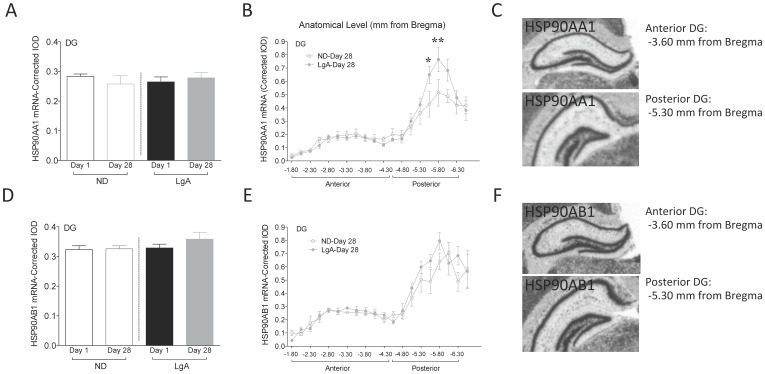
Stress-related gene expression in DG. (A) HSP90AA1 mRNA. (B) HSP90AA1 mRNA by anatomical level at Day 28 of cocaine withdrawal. *p<0.05; **p<0.01. (C) Representative X-ray image of HSP90AA1 mRNA in anterior and posterior DG. (D) HSP90AB1 mRNA. (E) HSP90AB1 mRNA by anatomical level at Day 28 of cocaine withdrawal. (F) Representative X-ray image of HSP90AB1 mRNA in anterior and posterior DG. Data are expressed as mean±SEM. Anterior (−3.60 mm from Bregma) and posterior (−5.30 mm from Bregma) DG.

Two stress candidate markers were selected based on recent data from our lab showing that over-expression of GR in mice forebrain increases HSP90AA1 and HSP90AB1 in DG [18]. For HSP90AA1, there were no significant effects of group or withdrawal time-point, and no interaction (Figure 4A). However, at Day 28, there was a significant effect of anatomical level (F_19,181_ = 27.51, p<0.001) and a group x anatomical level interaction (F_19,181_ = 1.807, p<0.05, Figure 4B). Specifically, there was an increase in HSP90AA1 mRNA in the posterior DG and this increase occurred to a greater extent in LgA rats (distance from Bregma: −5.55 mm, p<0.05; −5.80 mm, p<0.01). There was no effect of group, withdrawal time-point, or group x time-point interaction for HSP90AB1 mRNA levels (Figure 4D). At Day 28, there was a significant effect of anatomical level (F_19,188_ = 31.16, p<0.001), but no effect of group and no interaction (Figure 4E). There was no effect of anatomical level on Day 1 of withdrawal for either HSP90AA1 or HSP90AB1 mRNA levels (data not shown) and no significant differences observed in CA hippocampal subfields (Table 1).

### Correlations CORT-Stress-Related Genes

**Figure 5:**
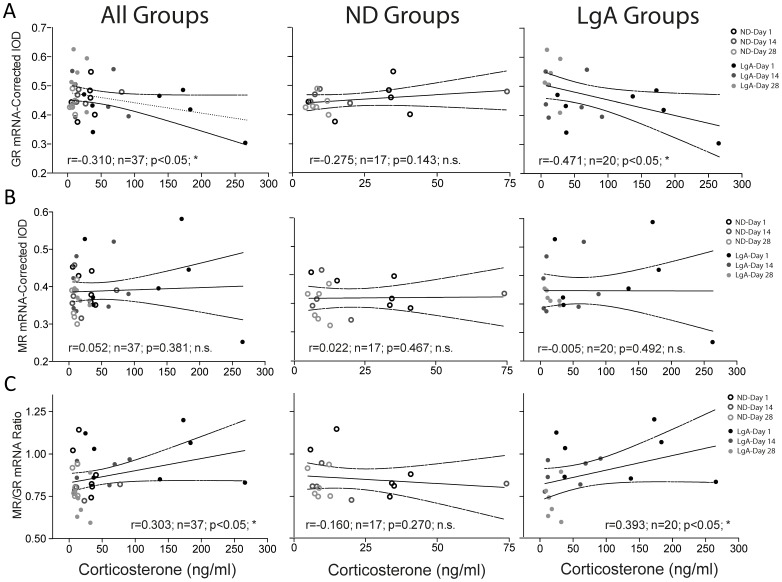
Correlation analysis. CORT levels correlated inversely with GR and positively with MR/GR mRNA expression in DG.

Correlation analyses revealed a negative correlation between CORT levels and DG GR mRNA (r = −0.310; n = 37; p<0.05; Figure 5A) in all rats, independent of treatment group or withdrawal time-point. If the analysis was conducted separately for each treatment group (i.e., basal effect in ND controls and drug effect in LgA groups), the correlation remained only for LgA groups (r = −0.471; n = 20; p<0.05). There was a positive correlation between CORT levels and DG MR/GR ratio (r = 0.303; n = 37; p<0.05; Figure 5C), which was driven by LgA groups (r = 0.383; n = 20; p<0.05). There were no significant correlations observed between CORT levels and DG MR mRNA (Figure 5B) or HSP90 mRNA (data not shown).

## Discussion

The current study uses an extended access model of cocaine SA, which elicits several features of human addiction (e.g., escalation of drug intake; [22]), to study whether changes in stress regulatory mechanisms in the hippocampus occur after prolonged withdrawal periods. The results suggest a role for the posterior hippocampus in the neural adaptations taking place during cocaine withdrawal, and point to HSP90AA1, a signaling partner of GR, as a novel target gene in the neurobiology of cocaine withdrawal. Specifically, we showed a delayed emergence of dysregulation of stress genes in posterior hippocampus following 28 days of withdrawal. This included increased GR mRNA in DG and CA3, increased MR and HSP90AA1 mRNA in DG, and decreased MR/GR mRNA ratio in DG and CA1. Moreover, CORT levels were increased 1 day following the last cocaine SA session but progressively decreased during the course of withdrawal. This time-dependent CORT decrease correlated negatively with GR mRNA and positively with MR/GR mRNA ratio in the DG.

It has been suggested that the long-lasting molecular neural adaptations mediated by cocaine exposure within the mesolimbic reward system are thought to underlie cocaine addiction and the propensity to relapse [3]–[5]. In fact, it has been shown that delayed drug-induced neurobiological effects over a two-month withdrawal period, likely contribute to sustained vulnerability to relapse [24]. For example, progressive increases in BDNF protein were observed after cocaine withdrawal (i.e., 30 to 90 days) within the VTA, accumbens and amygdala [2]. Interestingly, the present study reports an increase in GR, MR, and HSP90AA1 gene expression following 28 days of cocaine withdrawal in a region outside the classical reward pathway, the DG of hippocampus. In fact, gene expression changes (GR, MR, HSP90AA1) occur within the posterior (−4.52 to −6.80 mm from Bregma) hippocampal demarcation [25], a region known to receive many limbic projections, and one that has been implicated in ‘emotionality’ [26]–[27]. Moreover, these findings nicely complement work showing that dysphoria (i.e., increases in anxiety-like behavior) often accompanies drug withdrawal and can contribute to relapse [28], supporting the role of the hippocampus in anxiety-like behavior [29] and cocaine sensitization [18].

Hippocampal GR, as described above, is a mediator of the initial reactions to drugs of abuse [17], [18], and the present data suggests a role as a potential mediator of the long-term consequences of cocaine abuse (i.e., GR mRNA was increased in posterior DG and CA3 hippocampal subfield at Day 28 of withdrawal). In particular, these findings suggest a potential role of posterior hippocampus and GR in the neural adaptations following prolonged periods of withdrawal. Moreover, MR mRNA was increased in posterior DG, while MR/GR mRNA ratio was decreased in DG and CA1 hippocampal subfield at Day 28 of withdrawal. It has been suggested that a dysregulation of MR/GR balance brings CA1 neurons into a vulnerable state with consequences for regulation of the stress response [30].

Further, HSP90AA1 mRNA was increased in posterior DG following 28 days of withdrawal, while there was no change in HSP90AB1. Alterations in GR signaling partners such as HSP90AA1, provide further evidence for an interplay between the regulation of the stress system and withdrawal, and identify novel molecular candidates in mediating drug-induced neural plasticity in the hippocampus. HSP90 proteins are important molecular chaperones with two major cytosolic isoforms, HSP90AA1 (inducible form) and HSP90AB1 (constitutive form) [31]. HSP90 has been recently linked with numerous cellular pathways and is known to play a role in protein stabilization and adaptive responses to stress [32]. Our laboratory has reported that long-term GR over-expression in mice show increased level of HSP90AA1 gene expression as well as altered emotionality (i.e., increased anxiety-like behavior) and increased psychomotor sensitization to cocaine [18].

When evaluating the neuroendocrine response, CORT secretion was increased following the last cocaine administration, or Day 1 of withdrawal. However, CORT levels appeared to normalize during later withdrawal time-points (14 or 28 days). Similar results have been observed in human cocaine addicts who experienced elevated circulating diurnal cortisol levels 1 day following the last cocaine administration [33]. Although the effects of cocaine SA on plasma CORT have been previously characterized in rats [34]–[35], these effects appear to vary depending on the conditions under which cocaine is available [21]. For example, Mantsch and colleagues [21] observed that plasma CORT was increased immediately after each cocaine SA session, an effect that was correlated with the amount of cocaine intake. Previous experiments with a similar cocaine SA procedure (1 mg/kg/infusion, 6 h/sessions, 14 days) produced persistent changes in basal HPA function, including a reduction in basal CORT levels 21 days after the last SA session [36]. Our results suggest that the increase in CORT observed at Day 1 might be related to recent drug administration and therefore reflect direct and acute effects of cocaine on the HPA axis. The progressive increase in hippocampal GR mRNA could account for the observed reduction in basal CORT during the course of cocaine withdrawal. In fact, CORT regulation during the course of withdrawal correlated negatively with GR mRNA and positively with MR/GR mRNA ratio in DG. In agreement, increased hippocampal GR occupancy has previously been associated with a reduction in HPA axis activity [37], while lesioning or depletion of hippocampal GR resulted in CORT hypersecretion [38]. Although it has previously been suggested that an increase in GR protein in hippocampus might be responsible for reductions in basal CORT levels [21] following cocaine SA, another paper reported no change in dorsal hippocampus GR protein levels following cocaine SA [36]. Nonetheless, the balance in MR- and GR-mediated effects on stress system (i.e., CORT levels) is of critical importance to the set point of HPA activity and appears critical for neuronal excitability, stress responsibility, and behavioral adaptability [39].

Previous studies have focused on CORT as the primary index of HPA axis regulation or dysregulation. Yet circulating levels of glucocorticoids can return to normal, masking dysregulation at the neural level. The physiological consequences of sustained endocrine and neural alterations induced by a chronic stressor (e.g. chronic cocaine SA) can lead to permanently altered homeostasis of the system that comes at a biological price. This is the notion of ‘allostatic load’ put forth by McEwen [40]. Chronic cocaine dependence has been associated with allostatic changes in stress and reward pathways that are known to increase drug seeking in laboratory animals (see review in [41]). The present study shows that normalized CORT levels (i.e., Day 28) is clearly not an indicator that all has returned to normal in the brain, as significant changes emerge in gene regulation. What we find is evidence of an HPA system that has been reset, so once the immediate pressure has been removed to secrete CORT, significant and sustained changes in hippocampal gene regulation are uncovered (GR, MR, MR/GR ratio, HSP90AA1).

What are the potential implications of dysregulated hippocampal stress genes during cocaine withdrawal? The hippocampus is receiving increasing attention for its role in addiction and relapse [17], but is better known for its critical role in learning and memory [42], and in mediating the stress response [15]. In fact, combined exposure to cocaine and stress activate signaling pathways in the hippocampus crucial for physiological learning [43]. Moreover, the dorsal hippocampus and its interaction with the basolateral amygdala have been implicated in cocaine memory reconsolidation and subsequent context-induced cocaine-seeking behavior in rats [44]. Therefore, it is reasonable to speculate that the dysregulation of hippocampal stress genes that emerges during cocaine withdrawal (i.e., GR, MR, GR/MR ratio, HSP90AA1) could contribute to the aberrant engagement of learning mechanisms that underlie addiction [45]. Further investigation of the role of hippocampal stress genes in these processes is warranted.

In conclusion, extended daily access to cocaine activated the stress system at multiple levels and these changes occurred in a time-dependent manner. First, we observed elevated CORT levels 24 h after the last SA session. Our findings suggest that this immediate increase in CORT actually precluded evidence of molecular changes, perhaps due to enhanced negative feedback of the stress axis. As CORT levels receded, however, we observed sustained changes in stress-related genes (GR, MR, MR/GR ratio and HSP90AA1) in the posterior DG region of the hippocampus. Hippocampal GR is both a mediator of initial reactions to drugs of abuse [17], [18], and a potential regulator of the long-term neurobiological consequences of cocaine manifested during prolonged withdrawal. Taken together, these results suggest a role for posterior hippocampus, and specifically for GR in the prolonged neural adaptations that take place during cocaine withdrawal and may predispose an individual to drug-seeking behavior and relapse. Further, HSP90AA1 appears as a novel candidate target during prolonged cocaine withdrawal. Future investigations into the neurobiological mechanisms underlying cocaine withdrawal should pay particular attention to the hippocampus.
